# Sterile line Dexiang074A enhances drought tolerance in hybrid rice

**DOI:** 10.3389/fpls.2023.1054571

**Published:** 2023-03-09

**Authors:** Gengmi Li, Tao Zhang, Li Yang, Jian Qin, Qianhua Yang, Yingjiang Cao, Jing Luo, Xiangzhao Li, Lei Gao, Qian Chen, Xingping He, Yong Huang, Chuantao Liu, Ling He, Jiakui Zheng, Kaifeng Jiang

**Affiliations:** ^1^ Key Laboratory of Southwest Rice Biology and Genetic Breeding, Ministry of Agriculture/Luzhou Branch of National Rice Improvement Center, Rice and Sorghum Research Institute, Sichuan Academy of Agricultural Sciences, Deyang, China; ^2^ Biology and Molecular Biology Research Center, Rice and Sorghum Research Institute, Sichuan Academy of Agricultural Sciences, Deyang, China

**Keywords:** rice, drought stress, heterosis, hybrid rice, total soluble sugar content

## Abstract

Heterosis has been widely used in rice breeding, especially in improving rice yield. But it has rarely been studied in rice abiotic stress, including the drought tolerance, which is becoming one of the most important threaten in decreasing rice yield. Therefore, it is essential to studying the mechanism underlying heterosis in improving drought tolerance of rice breeding. In this study, Dexiang074B (074B) and Dexiang074A (074A) served as maintainer lines and sterile lines. Mianhui146 (R146), Chenghui727 (R727), LuhuiH103 (RH103), Dehui8258 (R8258), Huazhen (HZ), Dehui938 (R938), Dehui4923 (R4923), and R1391 served as restorer lines. The progeny were Dexiangyou (D146), Deyou4727 (D4727), Dexiang 4103 (D4103), Deyou8258 (D8258), Deyou Huazhen (DH), Deyou 4938 (D4938), Deyou 4923 (D4923), and Deyou 1391 (D1391). The restorer line and hybrid offspring were subjected to drought stress at the flowering stage. The results showed that Fv/Fm values were abnormal and oxidoreductase activity and MDA content were increased. However, the performance of hybrid progeny was significantly better than their respective restorer lines. Although the yield of hybrid progeny and restorer lines decreased simultaneously, the yield in hybrid offspring is significantly lower than the respective restorer line. Total soluble sugar content was consistent with the yield result, so we found that 074A can enhance drought tolerance in hybrid rice.

## Introduction

Rice is an important agricultural crop worldwide, and irrigated rice accounts for 70% of total rice production (Tiwari et al., 2020). It feeds more than half of the world’s population. As the human population surges, rice production will need to double by 2030 to meet global demand. Crop plants face a variety of abiotic stresses (You and Chan, 2015). Drought stress is one of the abiotic stresses that can inhibit crop growth and reduce crop yield in the field (Moore et al., 2009; Clark et al., 2013; Robles et al., 2018; Fu et al., 2019; Wang et al., 2019). To cope with climate change and environmental degradation, drought-tolerant rice varieties can be cultivated to reduce the risk of crop yield decline (Harris-Shultz et al., 2019). Because they are non-mobile organisms, it is important to coordinate genetic networks in rice to adapt to various biotic and abiotic stresses (Manhes et al., 2021; Song et al., 2023). For example, *STH1* encodes a negative regulator of salt tolerance in rice. Studies on *STH1* gene reveal a new mechanism for the synergistic regulation of salt tolerance and heading date in rice (Xiang et al., 2022). In *Arabidopsis*, sumoylation of NF-YC10 promotes the assembly of NF-YC complexes to enhance plant tolerance to high temperature stress (Huang et al., 2022). Among all abiotic stresses, drought stress is the most lethal because it can occur at any point in the rice plant life cycle, though it is especially damaging during another development (Guo et al., 2016). The phenomena of white anthers and sterile pollen grains finally caused yield loss in rice under water-deficit environments (Serraj et al., 2009). Drought stress may affect another compartment formation and microspore release by interfering with normal homeostasis of sugars, hormones, and reactive oxygen species (ROS) (Yu et al., 2019). Under drought stress conditions, many genes related to plant another development, including defective pollen wall (*DPW*) and tapetum degeneration deferred (*TDR*), are differentially expressed (Jin et al., 2013; Ma et al., 2017). However, the regulatory network that mediates the interaction between drought tolerance and fertility is complex and remains an obstacle to addressing their contradictions. Improving drought tolerance in rice without negatively affecting fertility remains a major challenge for rice breeders.

Under abiotic stress conditions, plants often sacrifice their fertility for survival. Therefore, fertility and stress resistance often oppose each other. In rice, drought tolerance may reduce seed fertility. In abiotic environments, the effects of drought on rice growth can be partly attributed to the accumulation of ROS (Halliwell, 2006). In this context, ROS generally refers to hydrogen peroxide (H_2_O_2_) and superoxide ( ${\text{O}}_{2}^{-}$ ), which can cause lipid peroxidation, protein degradation, nucleotide damage, and cell death (Anjum et al., 2011; Xu et al., 2016). In order to prevent oxidative damage, superoxide dismutase (SOD), catalase (CAT), peroxidase (POD), and ascorbic acid peroxidase (APX) scavenge active oxygen to reduce the impact of drought on cell oxidative damage and protect plant cells from active oxygen damage (Gill and Tuteja, 2010). Quantification of these enzymes is considered a general indicator of drought tolerance (Farooq et al., 2009; He et al., 2018). Malondialdehyde (MDA) is one of the products of cell membrane lipid peroxidation, and it can also aggravate cell membrane damage. The production of MDA can reflect the degree of membrane lipid peroxidation and indirectly reflect the antioxidant capacity of plant tissues (Sheoran et al., 2015). After drought stress, *Arabidopsis* and rice plants transformed with *sbWRKY30* genes and became tolerant to drought stress by changing the configurations of their root systems. Their proline content and their activities of SOD, POD, and CAT were higher than those of wild-type plants, and their MDA content was lower than that of wild-type plants. Gene *sbWRKY30* has a positive regulatory role in drought stress (Yang et al., 2020).

It is estimated that 50% of global rice production is affected by drought (Ren et al., 2019). The main nutrient in the rice grain is starch, with a content ranging from 81.23% to 92.73% (Omar et al., 2016). Starch is an (α-1,4)polyglucose-linked polymer. X-ray diffraction experiments showed that approximately 70% of the starch particles in rice were amorphous (mainly amylose), and the remaining 30% were amylopectin (Sajilata et al., 2006). Drought forcing during grain filling promoted plant senescence and redeployment of the carbon pool from vegetative tissues to grains (Yang et al., 2002), increased the grain filling rate, and shortened the grain filling period (Yang et al., 2001a). Sucrose is the main form of disaccharide transport in plants involved in photosynthate transport. In general, sucrose is transported by phloem to various organs (Eom et al., 2012; Ruan, 2012; De Schepper et al., 2013). Sucrose is not only the basis of physiological metabolism but also a signal molecule that plants use to coordinate physiological activities. It is the basic carbon skeleton monomer and energy source for seed formation and development, and it plays an important role in plant growth and seed development (Ruan et al., 2010; Ruan, 2012). It was previously reported that the transcription levels of *AtSWEET11*, *AtSWEET12*, and *AtSUC2* genes in *Arabidopsis* leaves were upregulated under drought stress conditions, resulting in enhanced response to sucrose export (Durand et al., 2016). Drought stress decreased photosynthetic carbon assimilation and seriously affected grain quality (Liu et al., 2017; Raed and Raju, 2018). In the study of soybean seed yield and drought, drought stress reduced the leaf photosynthesis rate, shoot biomass, and seed weight by 63.93%, 33.53%, and 41.65%, respectively. Drought stress increased the soluble sugar content, sucrose phosphosynthase content, and sucrose synthase and acid convertase activities; upregulated the expression of *GmSPS1*, *GmSuSy2*, and *GMA-INV*; and decreased the starch content by 15.13%. During seed development, drought stress increased the activities of sucrose synthesis and degradation enzymes, the expression levels of metabolism-related genes, and the expression levels of sucrose transporter genes during early seed development.

074B is a hybrid rice maintainer line with high combining ability as a parent, and 074A is the contra sterile line. 074B is used as a rice backbone parent line in Sichuan Province (Li et al., 2021). The hybrid offspring of 074A have efficient nitrogen utilization, high grain quality, and drought resistance. D4103 and D4727, two of its offspring, were recognized as super rice by the Ministry of Agriculture, China. Thus, 074A won the first prize in the Sichuan Province Science and Technology Progress Award. The effects of 17 cultivated rice varieties (074B, R146, D146, R727, D4727, RH103, D4103, R8258, D8258, HZ, DH, R938, D4938, R4923, D4923, R1391, and D1391) in southwest China on water stress tolerance in rice have been studied. In the present study, we used these 17 cultivars in a pot drought treatment experiment to make an assessment of the maintainer line 074B and to better promote the usage. These experiments were generally carried out during the later stage of rice growth (the main ear was exposed; i.e., the drought treatment was started 3–5 days before the beginning of the ear period) to allow the study of the mechanism underlying adaptation to water stress in 17 rice cultivars planted in southwest China. The research included the effects of water stress on the phenotype and yield of cultivated rice in southwest China, Fv/Fm, MDA, antioxidant enzyme activity, dry matter accumulation, and the percentage of dry matter in the panicle relative to the whole plant.

## Materials and methods

### Plant materials and growth conditions

The experiment was carried out at Fuyang Experimental Base at the China National Rice Research Institute from June to September 2020 and 2021, and the results showed the same trend in both years. The data collected in 2021 were used in this paper for analysis. Potting soil was used in the experiment. Each plastic bucket was 40 cm high and 30 cm in diameter, with 2 cm holes on the bottom. Rubber plugs in the bottom of the bucket were used for drainage and plugging during drought treatment. The organic matter content in the potting soil was 36.1 g/kg, total nitrogen 2.70 g/kg, total phosphorus 0.62 g/kg, total potassium 20.4 g/kg, alkali-hydrolyzed nitrogen 239 mg/kg, ammonium nitrogen 9.8 mg/kg, available phosphorus 24.1 mg/kg, and available potassium 62 mg/kg. The pH was 6.5, and each pot was loaded with 10 kg of dry soil. We used 074B as a maintainer line, 074A as a sterile line, and R146, R727, RH103, R8258, HZ, R938, R4923, and R1391 as restorer lines. The progenies were D146, D4727, D4103, D8258, HZ, D4938, D4923, and D1391 (**Table 1**). Rice was soaked at 37°C for 2 days and seed germination for 1 day and then sown 1 day later. After 30 days of seedling growth, the seedlings were transplanted into plastic buckets with six seedlings per pot and 20 pots per variety, including 10 pots for control treatment and 10 pots for drought stress treatment. All the pots were placed under a plastic rain shelter randomly. We kept the rice plants under normal watering conditions until the emergence of the first panicle. Then, the potted plants were divided into two groups. The control groups continued to have a shallow water layer. The rubber plugs of the 10 pots for drought stress groups were pulled out, and the water was drained out till the water potential at a soil depth of 20 cm dropped to −50 kPa. We then started the timer, waited another 48 h, and then started rehydration until the plants were mature and harvested.

**Table 1:** Genetic relationships between sterile, maintainer, restorer lines, and offspring.

Parents of three-line hybrid rice			Varieties (hybrid offspring)
Male sterile line	Maintainer line	Restorer lines	
Dexiang074A (074A)	Dexiang074B (074B)	Mianhui146 (R146)	Dexiangyou (D146)
		Chenghui727 (R727)	Deyou4727 (D4727)
		LuhuiH103 (RH103)	Dexiang4103 (D4103)
		Dehui8258 (R8258)	Deyou8258 (D8258)
		Huazhen (HZ)	Deyou Huazhen (DH)
		Dehui938 (R938)	Deyou 4938 (D4938)
		Dehui4923 (R4923)	Deyou 4923 (D4923)
		R1391	Deyou 1391 (D1391)

### Measurement of seed setting rate and dry matter weight

When the rice plants fully matured, the remaining three pots were harvested to determine the rice seed setting rate and yield per pot. The rice plants in each pot were separated into three parts for assessing dry weight: leaves, shoots and sheaths, and panicles. The total dry matter weight was calculated as the total weight of all three groups of parts, and the weight of panicles was calculated as panicle weight/the total dry matter weight.

### Fv/Fm measurement

Before rehydration, chlorophyll fluorescence was measured with a portable chlorophyll fluorescence spectrometer (PAM-2500 chlorophyll fluorescence system; Heinz Walz, Effeltrich, Germany) for the maximum chlorophyll fluorescence measurement. Rice flag leaves were wrapped in aluminum foil to simulate a dark environment, and the maximum fluorescence electron efficiency was measured after 30 min of dark treatment (Yu et al., 2020).

### Malondialdehyde measurement

Before rehydration, approximately 0.2 g of frozen leaves was homogenized in 2 ml of 5% trichloroacetic acid, and then 2 ml of 0.6% (m/v) thiobarbituric acid was added. The reaction mixture was placed in a boiling water bath for 30 min and then placed in cold water for rapid cooling. When the container reached room temperature, the absorbance of the mixture was measured at 450, 532, and 600 nm. The MDA content was calculated using the following formula: C = 6.45 × (A532 − A600) − 0.56 × A450 Dionisio-Sese and Tobita (Dionisio-Sese and Tobita, 1998).

### Measurement of antioxidant enzyme activities

Before rehydration, approximately 0.5 g of frozen spikelets was ground into a fine powder with the help of liquid nitrogen and then homogenized in 50 mM of sodium phosphate buffer (pH 7.0). The homogenate was centrifuged at 13,000 *g* for 15 min at 4°C, and the supernatant was stored in a refrigerator at −20°C for further use. The POD activity was measured following the method described by Chance and Maehly (1964), in which guaiacol was converted to tetraguaiacol and then monitored at 470 nm. CAT activity was measured according to a previously described method by Bergmeyer with some modifications (Bergmeyer et al., 1974). The CAT reaction mixture contained 25 mM of sodium phosphate buffer (pH 7.0) and 40 mM of H_2_O_2_ and was initiated by adding enzyme supernatant, and the change in the reaction solution in absorbance at 240 nm was recorded every 30 s for 2 min. One unit of CAT activity was defined as the amount capable of causing a change in absorbance of 0.01 unit/min.

### Measurement of total soluble sugar content

At the end of drought treatment, the panicles were sampled for assessment of total soluble sugar. Total soluble sugar content was determined using the anthrone-sulfate colorimetric method with some modifications (Dubois et al., 1956). A total of 0.5 g of panicle was mixed with 10 ml of ddH_2_O and boiled three times for a total of 30 min. After filtration, the extract was treated with anthrone, soaked in boiling water for 10 min, and cooled to room temperature, and the absorbance was measured at 620 nm with a spectrophotometer.

### Statistical analyses

SPSS 17.0 (SPSS, Chicago, IL, USA) was used for data analysis. A t-test was conducted to compare the differences between restorer lines and drought-stress plants. Variance (ANOVA) was conducted to compare the difference with a least significant difference (LSD) test at p = 0.05.

## Results

### Phenotype and yield statistics of progeny and restorer lines

**Figure 1:**
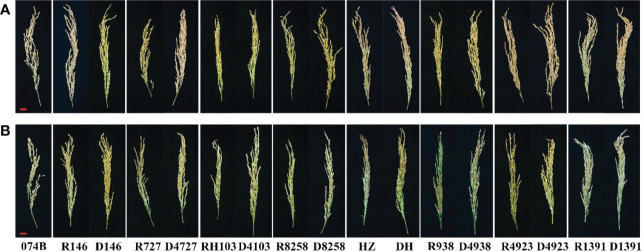
Rice panicles of progeny and restorer lines. **(A)** Panicles under control conditions. **(B)** Panicles under drought treatment conditions. Bar = 2 cm.

**Figure 2:**
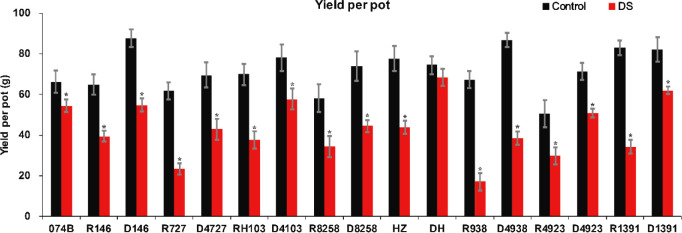
Rice yield per pot for 074B, restorer lines, and hybrid progeny. DS represents drought stress treatment. Error bars denote standard deviations (*n* = 3); “*” stands for significance between drought stress and control condition in the same cultivar (p ≤ 0.05).

In order to study the drought tolerance of the hybrid progeny of maintainer line 074B, we used 074B as a maintainer line, 074A as a sterile line, and R146, R727, RH103, R8258, HZ, R938, R4923, and R1391 as restorer lines. The progeny were D146, D4727, D4103, D8258, DH, D4938, D4923, and D1391. As shown in **Figure 1**, the seed setting rate decreased for both the control and hybrid offspring groups, but that of the control group decreased more seriously than that of the hybrid offspring group, indicating that drought caused more serious damage to the restorer line. The yield of maintainer line 074B decreased by 17.8% after drought treatment, while that of R146 and D146 decreased by 39.1% and 37.4%, respectively. The yield of R727 and its progeny D4727 decreased by 62.3% and 38.3%, respectively, while that of RH103 and D4103 decreased by 46% and 26%, respectively. HZ and its offspring DH decreased by 43.5% and 8.3%, respectively. The yield of R938 and progeny D4938 decreased by 74.6% and 55.7%, respectively; the yield of R4923 and D4923 decreased by 41.1% and 28.8%, respectively. The production of R1391 and offspring D1391 decreased by 58.7% and 24.6%, respectively. Compared with that of the control group, the yield of the offspring decreased less than that of the restorer groups. However, the yield of R8258 and its offspring D8258 decreased by 40.9% and 39.9%, respectively; the decrease in the yield of progeny was consistent with that of the restorer line D8258 (**Figure 2**). The data of the number of grains per panicle, number of panicles per basin, seed setting rate, 1,000-grain weight, and relative seed setting rate of 074B hybrid progeny can also fully explain how the 074B hybrid progeny has drought tolerance (**Supplementary Table 1**). The seed setting rate of R1391 decreased by 51.4%, which was twice as much as that of D1391 (25.2%). The number of grains per panicle (R727) and hybrid offspring (D4727) decreased by 25.6% and 0.3%, respectively. The 1,000-grain weight of R1391 and its progeny D1391 decreased by 2.4% and 0.07%, respectively. As shown, drought reduced the rice yield, but drought treatment conserved part of the rice yield of 074B hybrids to different degrees.

### Effects of drought on Fv/Fm and MDA content of progeny and restorer lines

**Figure 3:**
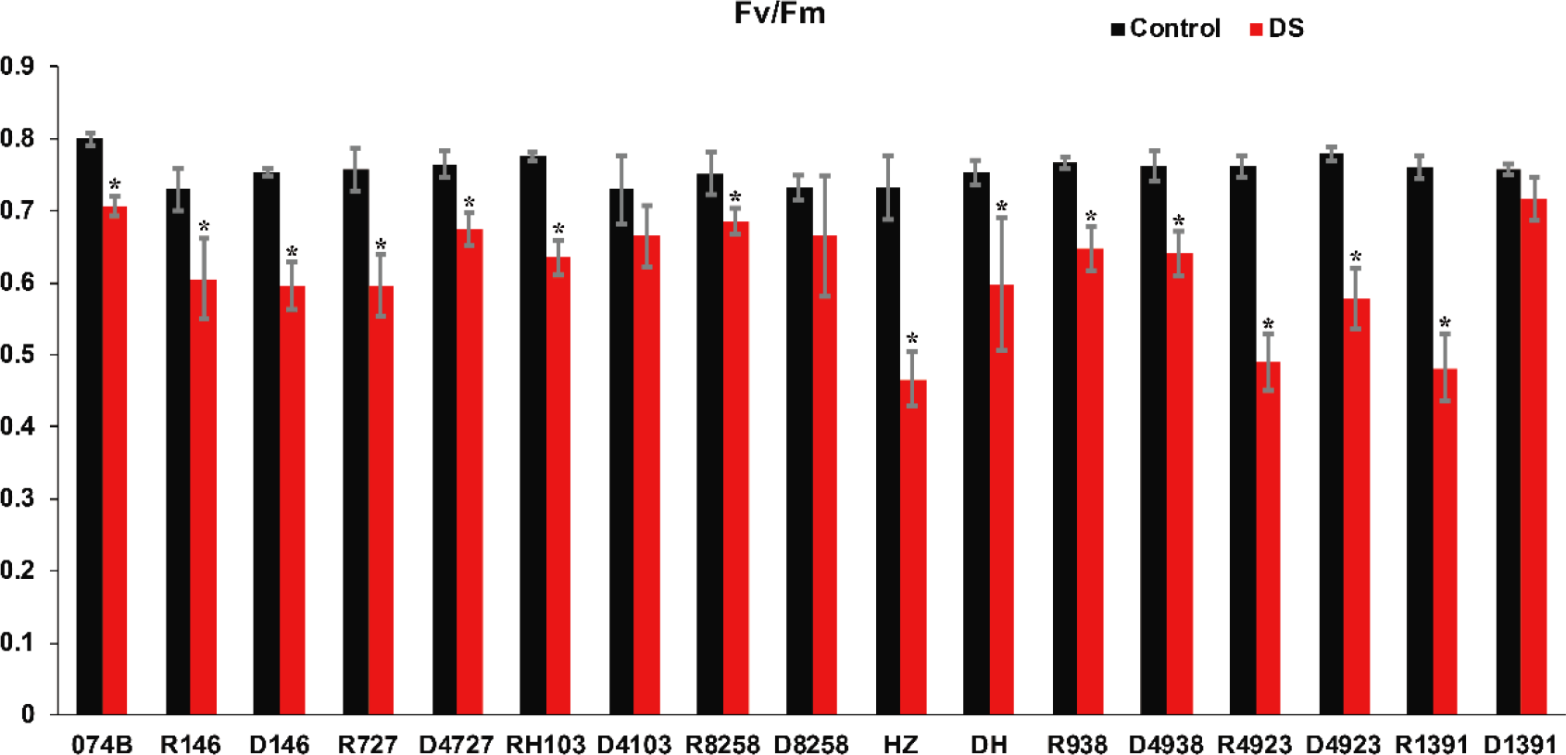
Fv/Fm value of restorer lines and hybrid progeny after drought treatment. Fv/Fm represents the maximum quantum efficiency of PSII; DS represents drought stress treatment. Error bars denote standard deviations (*n* = 3); “*” stands for significance between drought stress and control condition in the same cultivar (p ≤ 0.05).

In order to distinguish the drought resistance of hybrids and restorer lines, we measured Fv/Fm and MDA content. The higher the value, the lower the stress status of the plant, and the better its health status. The lower the value, the worse the health status of those under strong stress conditions. The results shown in **Figure 3** indicated that the Fv/Fm values of the male parent and hybrid offspring in the restorer line ranged from 0.7 to 0.8, indicating that the restorer line grew normally. After drought treatment, the Fv/Fm value of the maintainer line 074B decreased by 11.7%. The Fv/Fm values of the treatment group were lower than those of the restorer line; the Fv/Fm values of R727 and progeny D4727 decreased by 21.5% and 11.7%, respectively. The Fv/Fm values of RH103 and progeny D4103 decreased by 18% and 8.9%, respectively. The Fv/Fm values of HZ and its descendant DH decreased by 36.6% and 20.6%, respectively. The Fv/Fm values of R4923 and D4923 decreased by 35.8% and 26%, respectively. The Fv/Fm values of R1391 and D1391 decreased by 36.8% and 5.4%, respectively. Two sets of data showed no difference in Fv/Fm values between restorer lines and crosses. The Fv/Fm values of R8258 and D8258 decreased by 8.7% and 9.3%, respectively, and those of R938 and its descendant D4938 decreased by 15.7% and 15.7%, respectively. The Fv/Fm values of R146 and progeny D146 decreased by 17.3% and 21%, respectively. This was the only group of hybrid progeny whose Fv/Fm values showed that the restorer line was better at tolerating drought than its hybrid progeny. These results indicated that the parents and hybrid progeny were indeed affected by drought stress.

**Figure 4:**
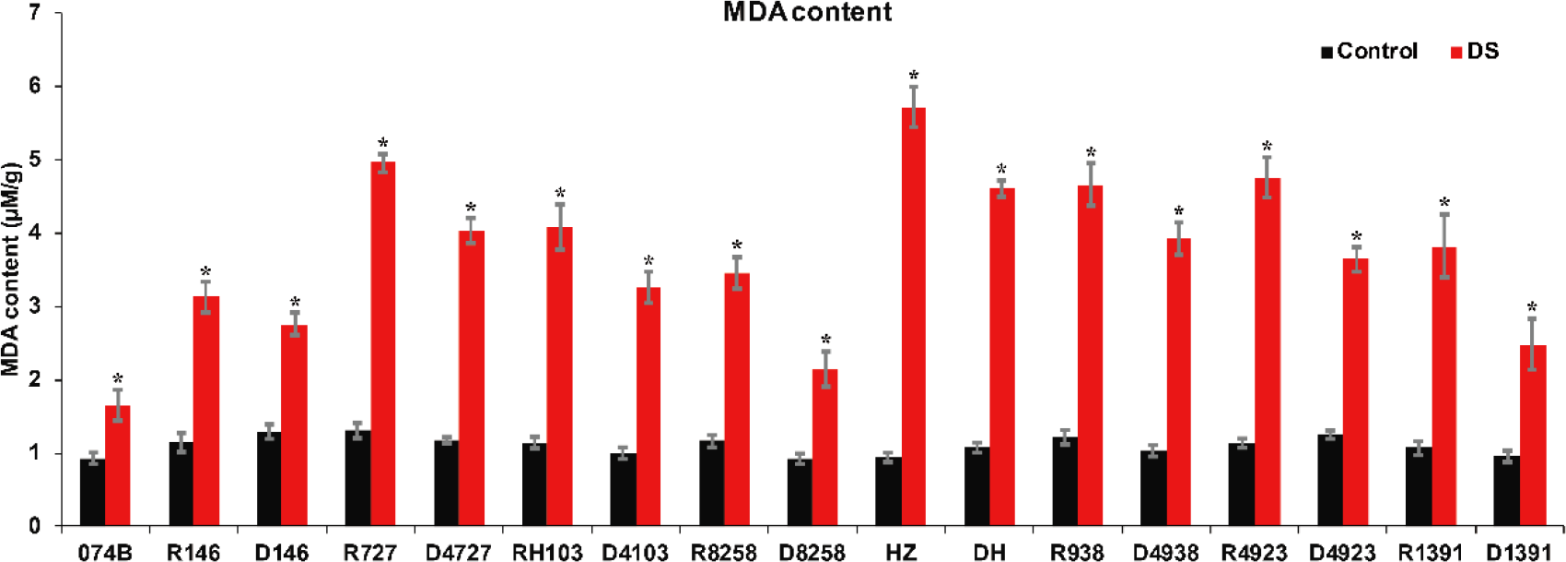
MDA content of restorer lines and hybrid progeny after drought treatment. MDA represents malondialdehyde; DS represents drought stress treatment. Error bars denote standard deviations (*n* = 3); “*” stands for significance between drought stress and control condition in the same cultivar (p ≤ 0.05).

MDA is the product of the disintegration of membrane lipid peroxidation, and it is an important parameter of the potential antioxidant capacity of plants. It can indirectly reflect the degree of tissue peroxidation damage. MDA content is a commonly used index in the study of resistance physiology. It can reflect the degree of stress damage for plants. To assess the drought resistance of 074B hybrid progeny, MDA content was measured in the spikes of restorer lines and hybrid progeny. The results showed that the MDA content of both restorer lines and hybrid progeny increased significantly after drought treatment. MDA content increased by 78.3% after drought treatment in 074B. After drought treatment, the MDA content of HZ and DH increased by 500.6% and 330.2%. R727 and progeny D4727 increased by 276.8% and 242.4%, respectively. The MDA content in RH103 and progeny D4103 increased by 256.9% and 225.6%, while the MDA content in R4923 and D4923 increased by 320.8% and 188.9%, respectively. Finally, for R1391 and offspring D1391, the content of MDA increased by 255.4% and 156.2% (**Figure 4**). Only one group showed no significant difference in MDA content—R938 and hybrid offspring D4938—which saw an increase of 281.3% and 277%, respectively. The significant increase in MDA content indicated that the parents and hybrid progeny were indeed affected by drought stress.

### Effects of drought on antioxidant enzyme activities in plants

**Figure 5:**
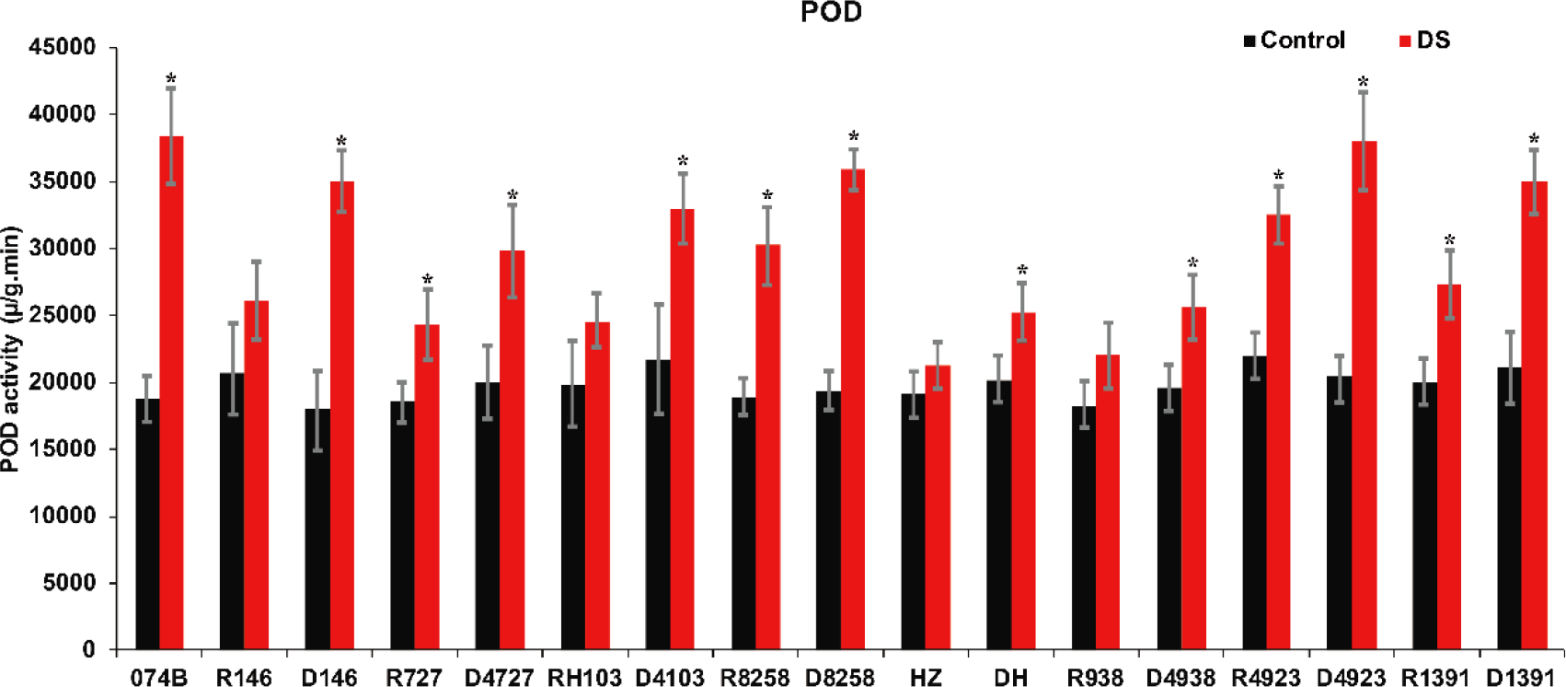
POD activity of restorer lines and hybrid progeny after drought treatment. POD represents peroxidase; DS represents drought stress treatment. Error bars denote standard deviations (*n* = 3); “*” stands for significance between drought stress and control condition in the same cultivar (p ≤ 0.05).

Plants contain a large number of peroxidases. These serve as markers of peroxisome, an oxidoreductase. Their activity changes constantly during plant growth and development, and POD activity can reflect the degree of stress damage to plants. In this study, POD activity was measured in the spikes of maintainer lines and hybrid progeny. The results shown in **Figure 5** indicated that POD activity in both parents and hybrids increased significantly after drought treatment. POD activity increased by 105% in 074B after drought treatment. After drought treatment, POD activity in R146 and progeny D146 increased by 26.4% and 94.2%, respectively, and for R727 and progeny D4727, POD activity increased by 31.4% and 48.7%, respectively. In RH103 and progeny D4103, POD activity increased by 23.7% and 52.2%, respectively. Therefore, the POD activity of the hybrid progeny was significantly higher than that of the restorer lines after drought stress, indicating that the parents and hybrid progeny were indeed affected by drought stress and that the hybrid progeny could better cope with the effects of drought stress.

**Figure 6:**
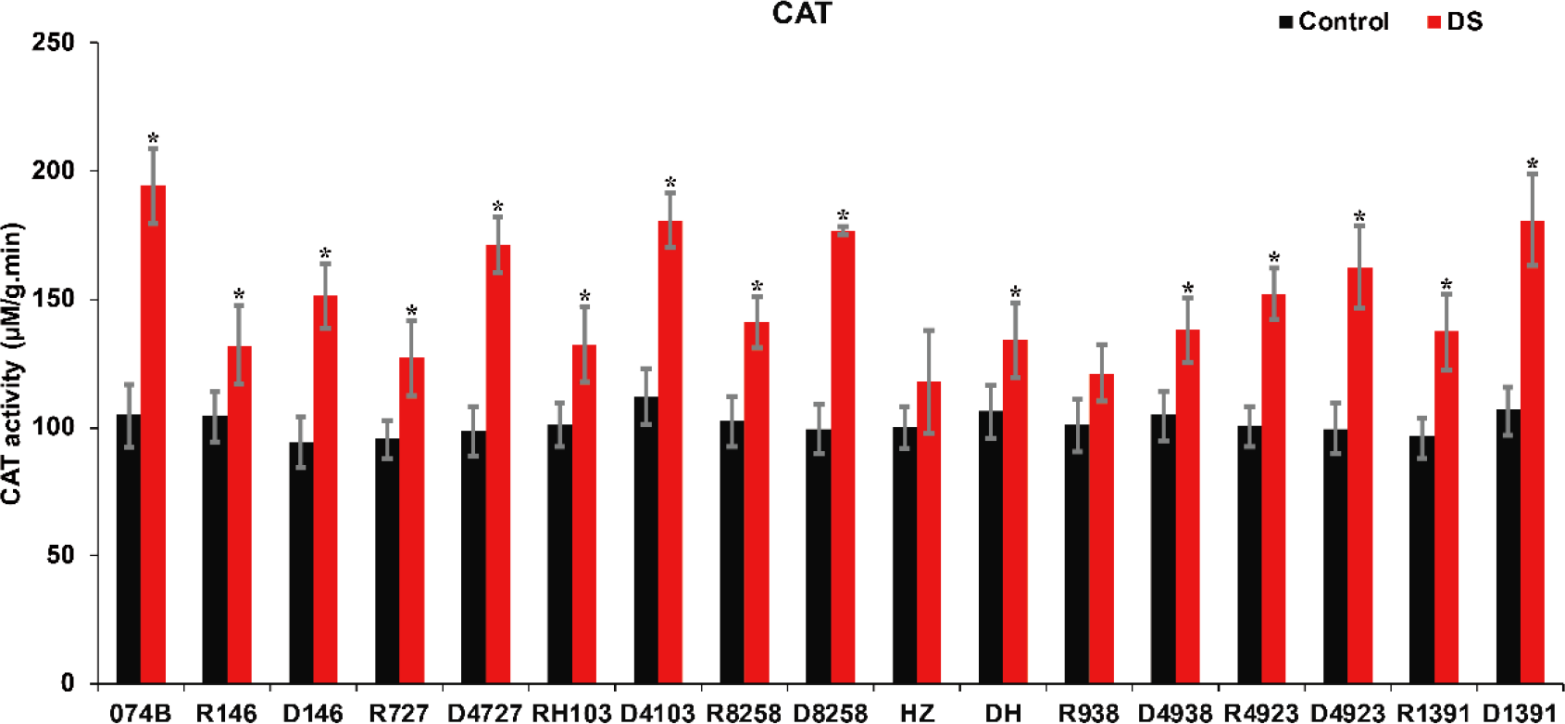
CAT activity of restorer lines and hybrid progeny after drought treatment. CAT represents catalase; DS represents drought stress treatment. Error bars denote standard deviations (*n* = 3); “*” stands for significance between drought stress and control condition in the same cultivar (p ≤ 0.05).

As a marker enzyme of peroxisomes, CAT is an oxidoreductase, and it can decompose H_2_O_2_ to produce molecular oxygen and water and remove hydrogen peroxide from plants to prevent it from poisoning the cells. CAT activity can reflect the degree of stress damage to plants. In this study, CAT activity was measured in the spikes of restorer lines and hybrid progeny. The results showed that the CAT activity of both parents and crosses increased significantly after drought treatment. CAT activity increased by 84.8% after drought treatment in 074B. After drought treatment, the CAT activity of R146 and progeny D146 increased by 26.3% and 60.9%, respectively. For R727 and progeny D4727, the value increased by 33.1% and 73.7%, respectively, while CAT activity increased by 30.8% and 61.0% in RH103 and D4103, respectively. The CAT activity in other restorer lines and their offspring also increased (**Figure 6**). This indicated that catalase activity was improved in restorer lines and their offspring. The CAT activity of hybrid progeny was significantly greater than that of the restorer line, indicating that plants of hybrid progeny were indeed more resistant to drought stress than the restorer line.

### Effect of drought on plant dry matter weight

**Figure 7:**
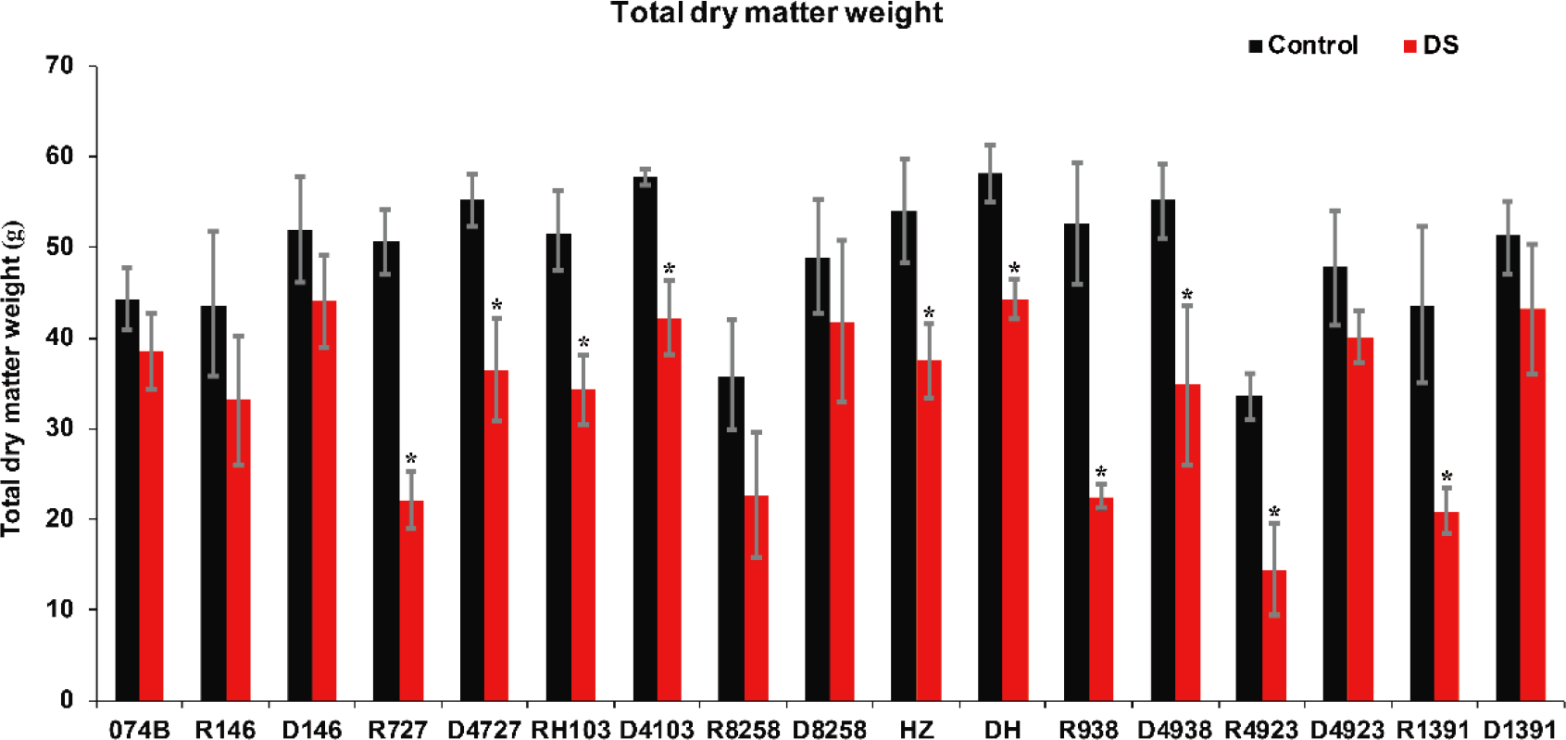
Total dry matter weight of restorer lines and hybrid progeny under drought stress treatment conditions. DS represents drought stress treatment. Error bars denote standard deviations (*n* = 3); “*” stands for significance between drought stress and control condition in the same cultivar (p ≤ 0.05).

Drought stress seriously affected the rate of accumulation of dry matter. During the flowering period, drought stress shortened the grain filling time, reduced the average and maximum rates of grain filling, and advanced the time at which the rate of grain filling peaked. It also affected the total amount of dry matter in plants. In this study, the degree of drought damage to plants was studied by measuring the amount of dry matter and the percentage of dry matter in the panicle relative to the whole plants in restorer lines and hybrid progeny. The results showed that the dry matter of restorer lines and hybrid progeny decreased significantly after drought treatment (**Figure 7**). After drought treatment, the amount of 074B dry matter decreased by 12.8%. The amount of dry matter in R146 and progeny D146 decreased by 23.9% and 15.1%, respectively. The dry matter of R727 and progeny D4727 decreased by 56.3% and 34.2%, respectively, as well as decreased by 33.5% and 27.0% for RH103 and progeny D4103, respectively. Similarly, the dry matter also showed a downward trend in the following comparisons: R8258 (36.8%) and progeny D8258 (14.2%), HZ (30.7%) and its progeny DH (24.0%), R938 (57.4%) and progeny D4938 (37.1%), and R4923 (57.1%) and progeny D4923 (16.4%). The amount of dry matter showed a downward trend after the plants were subjected to drought stress, and the amount of dry matter in hybrid progeny was significantly lower than in the restorer line, indicating that the hybrid progeny were indeed more tolerant to drought stress than the restorer line.

**Figure 8:**
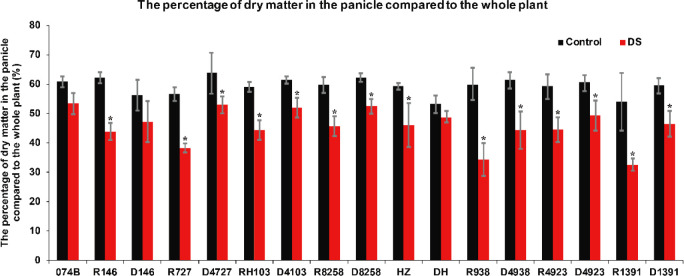
The percentage of dry matter in the panicle relative to the whole plant in the restorer lines and hybrid progeny after drought stress treatment. DS represents drought stress treatment. Error bars denote standard deviations (*n* = 3); “*” stands for significance between drought stress and control condition in the same cultivar (p ≤ 0.05).

Drought stress not only affected the rate of dry matter accumulation but also affected the rate of grain filling due to drought stress, which shortened the flowering period and affected the rate of grain filling. The amount of dry matter in the panicle was also affected. In this study, the percentage of dry matter in the panicle relative to the whole plant also confirmed that the plants were under drought stress. This study measured the percentage of dry matter in the panicle relative to the whole plant in restorer lines and hybrid progeny. The percentage of dry matter in the panicle relative to the whole plant in both restorer lines and hybrid progeny was significantly lower after drought treatment. The percentage of dry matter in the panicle relative to the whole plant decreased by 12.4% after 074B drought treatment. After drought treatment, the percentage of dry matter in the panicle relative to the whole plant between the restorer lines and progeny, R146 and D146, decreased by 29.5% and 16.0%, respectively. In R727 and progeny D4727, it decreased by 32.4% and 17.2%, respectively. In RH103 and progeny D4103, it decreased by 25.1% and 15.7%, respectively. In R8258 and its progeny D8258, it decreased by 23.5% and 15.5%, respectively. HZ and its progeny DH decreased by 22.3% and 8.3%, respectively. In R938 and progeny D4938, it decreased by 42.5% and 28.0%, respectively. In R4923 and progeny D4923, it decreased by 25.0% and 18.8%, respectively. In R1391 and progeny D1391, it decreased by 39.8% and 22.0%, respectively (**Figure 8**). The percentage of dry matter in the panicle relative to the whole plant showed a downward trend after drought stress, and the restorer line had significantly less dry matter than the hybrid progeny, indicating that the hybrid progeny were indeed more tolerant to drought stress than the restorer line.

### Effects of drought on total soluble sugar content in plants

**Figure 9:**
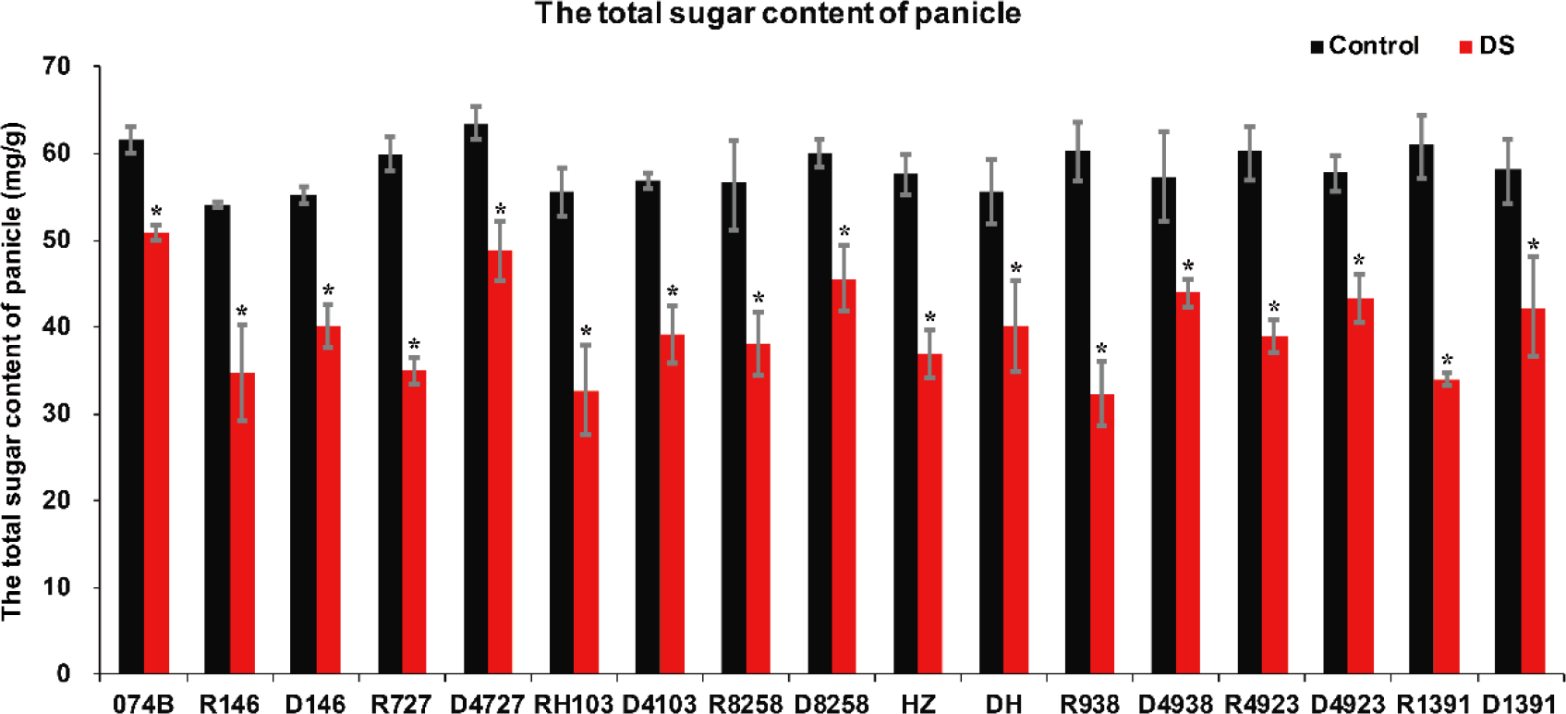
Total sugar content of restorer lines and hybrid progeny after drought stress treatment. DS represents drought stress treatment. Error bars denote standard deviations (*n* = 3); “*” stands for significance between drought stress and control condition in the same cultivar (p ≤ 0.05).

Drought stress decreased photosynthetic carbon assimilation and affected grain quality. In previous studies on soybean seeds and drought, drought stress reduced the rate of photosynthesis in the leaves; reduced shoot biomass and seed weight; increased soluble sugar content in the leaves, sucrose phosphate synthase, sucrose synthase, and acid convertase activities; and reduced starch content. The levels of starch, fructose, and glucose in seeds were decreased by drought stress during the later stages of grain filling. In this study, the index of the total sugar content of plants can also confirm that plants are under drought stress. In this study, the total sugar content of restorer lines and hybrid progeny was determined. The results showed that the total sugar content of restorer lines and hybrid progeny was significantly lower after drought treatment. The total sugar content of 074B decreased by 17.3% after drought treatment. After drought treatment, compared with the restorer line, the total sugar content in R146 and progeny D146 decreased by 35.5% and 25.0%, respectively, and in R727 and progeny D4727, the content decreased by 41.5% and 23.1%, respectively. The total sugar content decreased by 41.2% and 31.2% in RH103 and progeny D4103, respectively, while 46.4% and 23.2% total sugar content decreased in R8258 and progeny D8258, respectively. The total sugar content in other restorer lines and their progeny decreased in our results (**Figure 9**). The total sugar content showed a downward trend after the plants were subjected to drought stress, and the total sugar content of each restorer line was lower than that of its hybrid progeny, indicating that the hybrid progeny plants indeed had greater tolerance to drought stress than the restorer line.

## Discussion

Rice drought tolerance often has readily visible morphological, physiological, and biochemical characteristics as well as different genetic regulatory mechanisms (Yue et al., 2006). Although a large number of drought tolerance genes have been cloned in rice, marker-assisted selection breeding for drought tolerance has progressed slowly. Some factors have kept the progress slow: first, a considerable number of drought-tolerant genes have only been verified at the seedling stage and not at the reproductive stage. Second, these genes have little effect, so the drought tolerance of plants has not been significantly improved. Third, some genes have negative effects on plant growth, yield, or quality (Xia et al., 2019). Our present study showed that hybrids from Dexiang074A (074A) could significantly enhance yield, quality, and drought tolerance (**Figure 1**; Li et al., 2021).

It has been reported that many physiological indexes were used to evaluate the rice tolerance to drought stress such as Fv/Fm, MDA, and ROS (Wang et al., 2021). MDA is the product of membrane lipid peroxidation induced by ROS accumulation, often accumulated under stress conditions, which is consistent with our present study (**Figure 4**). There is a feedback mechanism of the ROS accumulation existing, which leads to an increase of antioxidant enzymes including POD and CAT (Mittler et al., 2022). In our present study, we found that the POD and CAT activities were increased under drought stress, while those of the hybrid offspring were higher than those of the restorer lines (**Figures 5**, **6**). Except for the ROS system, carbohydrate transportation and distribution are often affected by water conditions (Jing et al., 2022). Moderate drought stress can effectively promote the transport of non-structural carbohydrates stored in stems and sheaths to panicles (Yang et al., 2001b). Under normal conditions, the contribution of stem and sheath assimilates to yield is approximately 20%, and it can increase to 50% under drought-stress conditions. This phenomenon is closely related to stomata (Chaves et al., 2002). Because stomatal conductance and CO_2_ absorption are reduced under drought conditions, the generation of crop photosynthetic products is limited. Assimilates stored in stems and sheaths before grain filling become the main source of grain filling (Plaut et al., 2004). The total soluble carbohydrate decreased with an increase in moisture deficit stress level (Shatpathy et al., 2019). In our present study, we found that the total sugar content was decreased by drought stress, and the total sugar content of hybrid offspring was generally higher than that of restorer lines (**Figure 9**). Also, the percentage of dry matter in the panicle relative to the whole plant showed the same trend, decreased by drought stress and less in hybrid than in the respective restorer lines (**Figure 8**). Therefore, we concluded that the 074A used as a sterile line could improve the hybrid tolerance than the respective restorer lines.

Heterosis, including dominance, super dominance, and epistasis effects, refers to the phenomenon that progeny of diverse varieties of a species or crosses between species exhibit greater biomass, speed of development, and fertility than both parents (Liu et al., 2020). In breeding, researchers select higher relative water content, ear length, grain number per ear, biomass, leaf drying, and drought recovery rate to improve rice drought tolerance and yield and obtain new varieties with high yield and drought tolerance (Jabran et al., 2017). Parents with high drought tolerance screened by identification of drought response indices were used to develop drought-tolerant varieties (Ouk et al., 2006). Lafitte et al. (2006) initiated large-scale backcross breeding involving more than 160 parent varieties from 25 countries to improve drought tolerance. After drought treatment, several lines with significant changes in drought response were selected, and drought-tolerant lines were crossed with one to three recurrent parents to evaluate BC_2_F_2_ under drought conditions. Although drought stress results in a very low seed setting rate of backcross parents, many drought-tolerant backcross progeny lines produce seeds under drought stress, so they can provide drought-tolerant seed resources for rice-growing areas. Under drought stress during the reproductive period, directly selected seeds yielded 25% to 34% more than random lines. When assessed at similar levels of drought stress, it was noted that parent selection was particularly important in drought-tolerant breeding (Kamarudin et al., 2018). In addition, Verulkar et al. (2010) found that when severe drought stress occurred during the breeding period, selection leans toward greater gains under similar stress levels (yield reduction of 65% or greater under stress) than selection under non-stress conditions with no yield reduction under non-stress conditions (Verulkar et al., 2010). Recent studies indicated the grain yield of major quantitative trait loci (QTLs) with large effects (32%–33% variation) under drought conditions (Vikram et al., 2016; Fontes et al., 2021). In the present study, 074A was used as the female parent, eight hybrids were used as the research materials, and the results indicated that the hybrids’ yield was better than that of the restorer line parents’ (**Figure 2**). It has been reported that the F_1_ hybrid rice combination from a cross between 103S and IR17525 enhanced grain yield under drought stress (Cuong et al., 2014). This is consistent with our present study, which showed that the hybrid combination, with obvious heterosis in drought stress, generally performed better than the parents in dry matter, enzyme activity, yield, and other characteristics. In addition, the heterosis value of dry matter accumulation under drought stress is slightly decreased when compared with normal conditions. For dry matter accumulation, the heterosis value increased much more in the hybrids under drought pressure than under normal conditions. After the drought recovery, the dry matter accumulation of all hybrid offspring remained unchanged, indicating the potential of using 074A to produce drought-tolerant hybrids. The utilization of rice heterosis was generally carried out around the yield in China (Ouyang et al., 2022). Hybrid offspring of female parent 074A showed not only heterosis of yield but also enhanced drought tolerance. However, no similar studies on rice male parents have been reported. Therefore, the collection and creation of drought-resistant germplasm resources of rice female parents may be an effective way for drought-resistant breeding of high-yield hybrid rice.

## Conclusion

Dexiang074B (074B) and Dexiang074A (074A) here served as maintainer lines and sterile lines. Mianhui146 (R146), Chenghui727 (R727), LuhuiH103 (RH103), Dehui8258 (R8258), Huazhen (HZ), Dehui938 (R938), Dehui4923 (R4923), and R1391 served as restorer lines. The progeny were Dexiangyou (D146), Deyou4727 (D4727), Dexiang4103 (D4103), Deyou8258 (D8258), Deyou Huazhen (DH), Deyou 4938 (D4938), Deyou 4923 (D4923), and Deyou 1391 (D1391). 074B, restorer lines, and offspring were used for drought stress treatment; the results of the study found that compared with the restorer lines, the offspring of 074A sterile lines in all in the determination of drought index have excellent performance. The decline in the yield was less pronounced for the offspring of 074A than for the restorer line. These hybrids are generally drought tolerant. Currently, the most concerning issue is the yield of crops under various stress conditions. The yield traits D4727 and DHZ showed the best performance, indicating that these two varieties have the least impact on yield under low-water conditions. This method can provide ideas for breeding for different stress conditions such as drought and low and high temperatures. The results of other drought tolerance indexes also showed that 074A progeny exhibited considerable drought tolerance. However, the yield traits of the 074A offspring showed excellent performance alongside drought resistance, indicating that this breeding method can allow the plant to cope with severe environmental changes, indicating that 074A may contain drought resistance genes and other novel genes with drought functions.

## Data availability statement

The original contributions presented in the study are included in the article/**Supplementary Material**. Further inquiries can be directed to the corresponding authors.

## Author contributions

Data curation: JQ, YC, and QY. Funding acquisition: GL, JZ, KJ, and TZ. Investigation: JL, XL, QC, XH, YH, CL, and LH. Writing the original draft: GL, TZ, and LY. Writing—review and editing: KJ, JZ, and GL. All authors contributed to the article and approved the submitted version.
